# Mismatch repair protein and *MLH1* methylation status as predictors of response to adjuvant therapy in endometrial cancer

**DOI:** 10.1002/cam4.3691

**Published:** 2021-01-15

**Authors:** Mikko Loukovaara, Annukka Pasanen, Ralf Bützow

**Affiliations:** ^1^ Department of Obstetrics and Gynecology Helsinki University Hospital and University of Helsinki Helsinki Finland; ^2^ Department of Pathology Faculty of Medicine Helsinki University Hospital and Research Program in Applied Tumor Genomics University of Helsinki Helsinki Finland

**Keywords:** endometrial cancer, microsatellite instability, mismatch repair protein, *MLH1* methylation, p53, *polymerase ϵ*

## Abstract

**Background:**

Mismatch repair (MMR) system has been implicated in the response of mammalian cells to ionizing radiation and DNA damaging agents. We investigated the value of the MMR system in predicting response to adjuvant therapy in endometrial cancer.

**Methods:**

This was a single institution retrospective study. MMR protein status of endometrial carcinomas was assessed by immunohistochemistry. MMR deficient (MMR‐D) tumors were identified as *MLH1* methylated or nonmethylated by methylation‐specific multiplex ligation‐dependent probe amplification. Tumors with abnormal p53 staining or *polymerase ϵ* exonuclease domain mutation were excluded from the MMR proficient subgroup, which was termed as “no specific molecular profile” (NSMP). Disease‐specific survival analyses were adjusted for age, stage, histology and grade, depth of myometrial invasion, and lymphovascular space invasion.

**Results:**

A total of 505 patients were included in the study. Median follow‐up time was 81 months (range 1–136). Whole pelvic radiotherapy (adjusted hazard ratio [HR] 0.092 vs. no adjuvant therapy) and chemotherapy combined with radiotherapy (adjusted HR 0.18) were associated with improved disease‐specific survival in the NSMP subgroup (n = 218). In contrast, adjuvant therapies showed no effect on disease‐specific survival in the full MMR‐D cohort (n = 287) or in *MLH1* methylated tumors (n = 154). Whole pelvic radiotherapy (adjusted HR 25 vs. no adjuvant therapy/vaginal brachytherapy) and chemotherapy combined with whole pelvic radiotherapy (adjusted HR 32) were associated with poor disease‐specific survival in MMR‐D nonmethylated tumors (n = 70).

**Conclusion:**

MMR protein and *MLH1* methylation status predict the response to adjuvant therapy in endometrial cancer. The MMR system could be utilized for selection of patients who most likely benefit from adjuvant therapy.

## Graphical abstract

Adjusted for the most important clinicopathologic risk factors, adjuvant therapies were associated with improved disease‐specific survival in MMR proficient endometrial cancers, defined as those with no specific molecular profile. Adjuvant therapies showed no effect on survival in the full cohort of MMR deficient tumors, but were associated with poor survival in MMR‐D nonmethylated tumors.

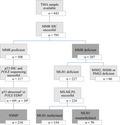

## 1 INTRODUCTION

The mismatch repair (MMR) system is a highly conserved DNA repair mechanism that corrects mismatched base pairs generated during DNA replication. A deficient MMR system is characterized by high levels of microsatellite instability (MSI) across the genome, leading to a large somatic mutational burden in MMR deficient cells.^1^ The normal function of the MMR system is dependent on four key enzymes: MLH1, MSH2, MSH6, and PMS2. MMR protein loss by immunohistochemistry can be used as a marker for MSI. Alternatively, MSI may be directly detected after amplification of microsatellite markers with methods based on polymerase chain reaction.

About 30% of endometrial cancers exhibit MMR deficiency.^2^ These are mostly sporadic cases in which MMR deficiency usually results from hypermethylation of the *MLH1* promoter. About 3% of endometrial cancers are associated with Lynch syndrome, that is, inherited mutation in one of the MMR genes.^3^

MSI is a characteristic signature of one of the four molecular subgroups of endometrial cancer described by The Cancer Genome Atlas (TCGA).^4^ The microsatellite unstable hypermutated subgroup is associated with an intermediate prognosis, similar to the copy‐number low subgroup, in contrast to the *polymerase ϵ* (*POLE*) ultramutated subgroup and the copy‐number high subgroup that are associated with an excellent outcome and poor outcome, respectively.

Development of classifiers that recapitulate the prognostic subgroups of TCGA without the need for costly and labor‐intensive genomic methodology provided tools for a surrogate TCGA approach in clinical practice.^5^, ^6^ The surrogate classifiers are based on the combination of MSI analysis and/or MMR protein immunohistochemistry, *TP53* mutational testing and/or p53 immunohistochemistry, and *POLE* mutational analysis.

Several in vitro studies have implicated MMR system in the response of mammalian cells to ionizing radiation and DNA damaging agents.^7^, ^8^ The role of MMR in response to ionizing radiation is yet controversial, but available preclinical data suggest that tumors containing a significant fraction of cells deficient in MMR will demonstrate reduced responsiveness to specific drugs. However, it has not been unequivocally defined whether MMR protein status influences the efficacy of adjuvant therapy in endometrial cancer. The objective of this study was to determine the value of MMR protein status in predicting response to different adjuvant therapies in a retrospective cohort of patients with endometrial cancer that were classified into molecular subgroups by immunohistochemistry of MMR proteins and p53, and sequencing of *POLE*. In addition, we studied *MLH1* methylation status as a predictor of treatment response in MMR deficient (MMR‐D) endometrial cancers.

## 2 MATERIALS AND METHODS

### 2.1 Study population and data collection

This was retrospective study of patients who underwent surgical treatment for stage I–IV endometrial carcinoma at the Department of Obstetrics and Gynecology, Helsinki University Hospital, between 1 January 2007 and 31 December 2012. Clinicopathologic data were abstracted from institutional medical and pathology records. Stage was determined according to the International Federation of Gynecology and Obstetrics guidelines revised in 2009.^9^ The cutoff for age was based on the finding that age >65 years is an independent poor prognostic factor in endometrial cancer.^10^ Lymphovascular space invasion was defined as the presence of adenocarcinoma, of any extent, in endothelium‐lined channels of uterine specimens outside the tumor.

Disease‐specific survival was calculated as the time from surgery to death from endometrial cancer. Cause of death was mainly based on medical records. Missing data were complemented from death certificates derived from Statistics Finland.

Standard surgery included total hysterectomy and bilateral salpingo‐oophorectomy. Lymphadenectomy was performed in selected patients. Adjuvant therapy was tailored according to stage and histologic findings at surgery. Patients with early stage endometrioid carcinoma with high‐risk features generally received either vaginal brachytherapy or whole pelvic radiotherapy. Vaginal brachytherapy was mainly limited to patients in whom surgical nodal assessment was performed. Patients with nonendometrioid or advanced stage endometrioid carcinoma received multimodality treatment with chemotherapy and radiation. Paclitaxel/carboplatin doublet was the standard chemotherapy regimen. The study followed the reporting recommendation of tumor marker studies (REMARK) guidelines.^11^ It was approved by the Institutional Review Board and the National Supervisory Authority for Welfare and Health.

Chest radiograph and upper abdominal ultrasound were the routine imaging studies for the detection of distant metastases preoperatively. Local disease spread was assessed by vaginal ultrasound and, as of January 2012, also by pelvic magnetic resonance imaging.

### 2.2 Molecular classification

A tissue microarray (TMA) was constructed on primary tumor samples as previously described.^12^ The following monoclonal antibodies were used for chromogenic immunohistochemistry on multicore TMA slides: MLH1 (ES05, Dako), MSH2 (G219‐1129, BD Biosciences), MSH6 (EPR3945, Abcam), PMS2 (EPR3947, Epitomics), and p53 (DO‐7, Dako). TMA slides were scanned with a three‐dimensional Histech Pannoramic 250 Flash II scanner by Fimmic Oy. Slide images were managed and analyzed with WebMicroscope Software (Fimmic Oy). MMR deficiency was defined as a complete loss of nuclear expression in carcinoma cells of one or more MMR proteins (MLH1, MSH2, MSH6, and PMS2). MMR proficiency was defined as the presence of normal staining of all MMR proteins. Abnormal p53 staining was defined as strong and diffuse nuclear staining or completely negative staining in carcinoma cells. Weak and heterogeneous staining was classified as wild‐type expression. Stromal cells and inflammatory cells served as internal control for MMR protein and p53 stainings.

*POLE* exonuclease domain mutation (EDM) screening of hot spots in exons 9, 13, and 14 was performed by direct sequencing.^13^ Only samples with high‐quality sequence for all of the four *POLE* hot spots examined were included in the study. Conforming to the Proactive Molecular Risk Classifier for Endometrial Cancer (ProMisE),^5^ we excluded from MMR proficient cases those that showed aberrant p53 (p53 abn) staining or *POLE* EDM, and termed this subgroup as “no specific molecular profile” (NSMP). The NSMP subgroup can also be referred to as p53 wild type (p53 wt).^5^ p53 abn and NSMP/p53 wt correspond to the copy‐number high and copy‐number low subgroup, respectively, in the TCGA classification system.^5^, ^6^

### 2.3 Methylation‐specific multiplex ligation‐dependent probe amplification

Methylation‐specific multiplex ligation‐dependent probe amplification (MS‐MLPA) was performed on MLH1 deficient tumors to evaluate *MLH1* promoter methylation levels in Deng promoter regions C and D. We used the SALSA MMR MS‐MLPA Kit ME011 (MRC Holland) on 250 ng of DNA from each sample. All MS‐MLPA reactions, analyses and calculations of methylation dosage ratios were done according to the manufacturer's instructions. MS‐MLPA products were separated by capillary electrophoresis on ABI 3730 Automatic DNA sequencer (Applied Biosystems) and analyzed using GeneMapper 5.0 genotyping software (Applied Biosystems). To calculate the methylation ratio, each peak height from HhaI‐digested tumor DNA was divided by its corresponding peak height from the undigested tumor DNA. To compensate for differences in polymerase chain reaction efficiency of the individual samples, each peak height (digested and undigested) was normalized dividing the probe amplification product by the average value of the control probes without an HhaI enzyme site. According to the manufacturer's recommendations, the hypermethylation threshold is defined as the mean methylation dosage ratio in reference samples (from healthy patients) plus two standard deviations. Since our reference samples did not present methylation in the abovementioned regions, the technical threshold of 0.15 was used as a cutoff.^14^ Tumors with a methylation ratio >0.15 (corresponding to 15% of methylated DNA) in region C and/or region D were considered hypermethylated.

### 2.4 Statistical methods

Chi‐squared test was used for comparison of categorical variables, and analysis of variance and Kruskal–Wallis test for comparison of continuous variables after testing for normality by Shapiro–Wilk test. Survivals were estimated using univariable and multivariable Cox regression analyses. Statistical significance was set at *p* < 0.05. Data were analyzed using the Statistical Package for the Social Sciences version 25 software (IBM Corp.).

## 3 RESULTS

**FIGURE 1:**
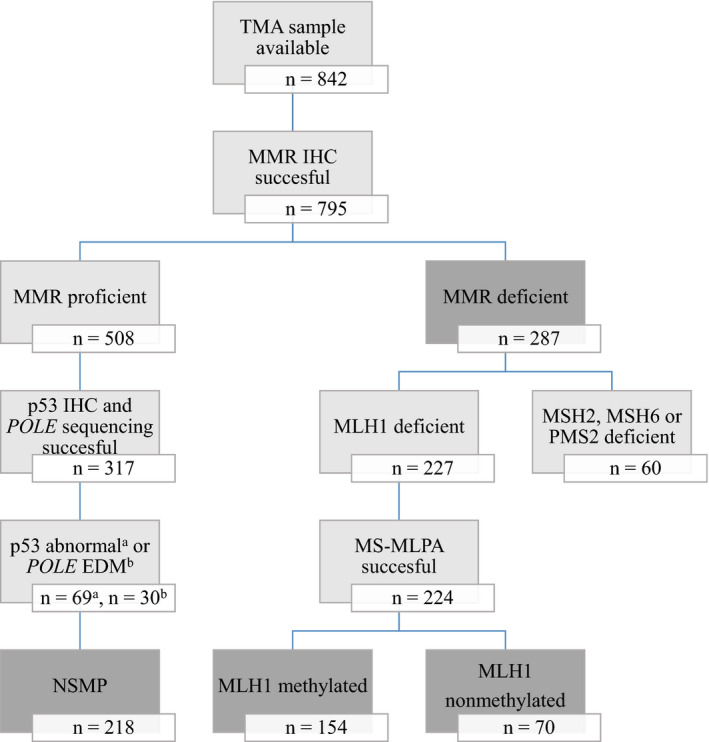
Flow chart depicting patient selection for the study. EDM, exonuclease domain mutation; IHC, immunohistochemistry; MMR, mismatch repair; MS‐MLPA, methylation‐specific multiplex ligation‐dependent probe amplification; NSMP, no specific molecular profile; *POLE*, *polymerase ϵ*; TMA, tissue microarray

Seven hundred ninety‐five patients with a TMA sample available were eligible for the study (Figure 1). MMR deficiency was identified in 287 tumors and MMR proficiency in 508 tumors. Of the MMR proficient tumors, p53 immunohistochemistry and *POLE* sequencing were successful in 317. After excluding tumors with abnormal p53 staining (n = 69) or *POLE* EDM (n = 30), 218 patients were included in the NSMP subgroup. MS‐MLPA was successfully carried out on 224 MLH1 negative tumor samples in the MMR‐D subgroup. Median follow‐up time was 81 months (range 1–136).

Clinicopathologic data for the MMR‐D and NSMP subgroups are shown in Table 1. Baseline characteristics were balanced between subgroups with the exception of older age, higher rate of pelvic‐aortic lymphadenectomy and lower proportion of well‐differentiated endometrioid carcinomas in the MMR‐D subgroup.

**TABLE 1:** Characteristics of the study population according to molecular subgroup

	MMR‐D (n = 287)	NSMP (n = 218)	*p*
Age (years) (median [interquartile range])	70 (61–77)	66 (60–73)	0.002**
Body mass index (kg/m^2^) (median [interquartile range])	27.3 (23.5–32.5)	28.5 (24.3–33.2)	0.050
Pelvic lymphadenectomy	165 (57.5%)	129 (59.2%)	0.704
Pelvic‐aortic lymphadenectomy	50 (17.4%)	19 (8.7%)	0.005**
Stage			0.110
IA	140 (48.8%)	123 (56.4%)	
IB	66 (23.0%)	42 (19.3%)	
II	22 (7.7%)	23 (10.6%)	
IIIA	19 (6.6%)	9 (4.1%)	
IIIB	4 (1.4%)	1 (0.5%)	
IIIC1	24 (8.4%)	13 (6.0%)	
IIIC2	8 (2.8%)	1 (0.5%)	
IVA	0 (0%)	0 (0%)	
IVB	4 (1.4%)	6 (2.8%)	
Histology			0.791
Endometrioid carcinoma	264 (92.0%)	206 (94.5%)	
Clear cell carcinoma	7 (2.4%)	5 (2.3%)	
Serous carcinoma	4 (1.4%)	2 (0.9%)	
Carcinosarcoma	4 (1.4%)	2 (0.9%)	
Undifferentiated carcinoma	8 (2.8%)	3 (1.4%)	
Grade (for endometrioid only)			0.000002***
1	127 (48.1%)	141 (68.4%)	
2	83 (31.4%)	52 (25.2%)	
3	54 (20.5%)	13 (6.3%)	
Myometrial invasion ≥50%	120 (41.8%)	83 (38.1%)	0.396
Lymphovascular space invasion	80 (27.9%)	49 (22.5%)	0.168
Adjuvant therapy			0.217
VBT	129 (44.9%)	116 (53.2%)	
WPRT	47 (16.4%)	28 (12.8%)	
Chemotherapy	10 (3.5%)	7 (3.2%)	
Chemotherapy and VBT	20 (7.0%)	10 (4.6%)	
Chemotherapy and WPRT	46 (16.0%)	24 (11.0%)	

Abbreviations: MMR‐D, mismatch repair deficient; NSMP, no specific molecular profile; VBT, vaginal brachytherapy; WPRT, whole pelvic radiotherapy.

** *p* < 0.01.

*** *p* < 0.001.

Of the 505 patients in the final study population, 363 (71.9%) underwent pelvic or pelvic‐aortic lymphadenectomy (Table 1). Of those considered to be at risk for lymph node involvement as per the most recent guidelines by the European Society for Medical Oncology (ESMO), European Society of Gynaecological Oncology (ESGO), and European Society for Radiotherapy and Oncology (ESTRO),^15^ that is, those with grade 3 endometrioid carcinoma, nonendometrioid carcinoma or grade 1–2 carcinoma with deep (≥50%) myometrial invasion, 75.6% (180/238) underwent pelvic or pelvic‐aortic lymphadenectomy. Of those at low risk for lymph node involvement,^15^ 68.5% (183/267) underwent lymphadenectomy. The distribution of adjuvant therapies across postoperative ESMO‐ESGO‐ESTRO risk groups^16^ is shown in Table S1.

**FIGURE 2:**
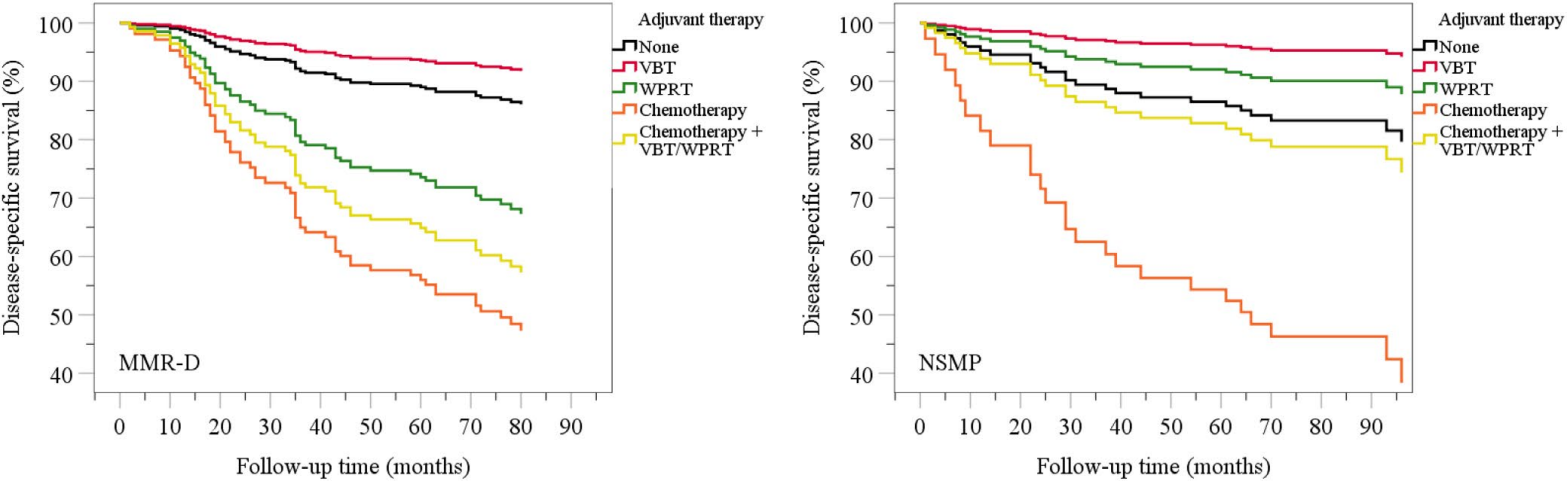
Cox regression disease‐specific survival plots for (A) mismatch repair deficient (MMR‐D) and (B) “no specific molecular profile” (NSMP) subgroups. VBT, vaginal brachytherapy; WPRT, whole pelvic radiotherapy

Univariable Cox regression analyses are shown in Table 2. The corresponding Cox regression plots are shown in Figure 2. Chemotherapy with or without radiotherapy was associated with an increased risk of disease‐related death in the MMR‐D subgroup. Vaginal brachytherapy was associated with a decreased risk of disease‐related death in the NSMP subgroup.

**TABLE 2:** Univariable Cox regression disease‐specific survival analyses for MMR‐D and NSMP subgroups

	MMR‐D (n = 287)			NSMP (n = 218)		
	N	HR (95% CI)	*p*	N	HR (95% CI)	*p*
Adjuvant therapy			0.000003***			0.001**
None	35	1		33	1	
VBT	129	0.57 (0.18–1.8)	0.344	116	0.26 (0.080–0.87)	0.029*
WPRT	47	2.6 (0.87–8.0)	0.087	28	0.57 (0.14–2.4)	0.446
Chemotherapy	10	5.0 (1.2–20)	0.023*	7	4.2 (1.0–18)	0.050
Chemotherapy and VBT/WPRT	66	3.7 (1.3–11)	0.014*	34	1.3 (0.42–4.0)	0.645

Abbreviations: CI, confidence interval; HR, hazard ratio; MMR‐D, mismatch repair deficient; NSMP, no specific molecular profile; VBT, vaginal brachytherapy; WPRT, whole pelvic radiotherapy.

* *p* < 0.05.

** *p* < 0.01.

*** *p* < 0.001.

To assess the independent effect of adjuvant therapy on patient outcome, we performed multivariable Cox regression analyses of disease‐specific survival (Table 3). The effect of adjuvant therapy on survival was not significant in the MMR‐D subgroup when controlling for age, stage, and uterine risk factors. Deep (≥50%) myometrial invasion and lymphovascular space invasion were identified as independent predictors of poor outcome. In contrast, adjuvant therapy showed an independent effect on disease‐specific survival in the NSMP subgroup, along with histologic subtype and lymphovascular space invasion. Specifically, whole pelvic radiotherapy and chemotherapy combined with radiotherapy were associated with improved outcome.

**TABLE 3:** Multivariable Cox regression disease‐specific survival analyses for MMR‐D and NSMP subgroups

	MMR‐D (n = 287)			NSMP (n = 218)		
	N	HR (95% CI)	*p*	N	HR (95% CI)	*p*
Age >65 years	180	1.4 (0.81–2.6)	0.219	116	1.7 (0.68–4.3)	0.256
Stage II–IV	81	1.4 (0.68–2.8)	0.372	53	3.8 (0.79–18)	0.096
Histology			0.567			0.001**
Endometrioid grade 1–2	210	1		193	1	
Endometrioid grade 3	54	1.4 (0.74–2.6)	0.299	13	9.8 (2.9–33)	0.0002***
Nonendometrioid	23	1.1 (0.45–2.5)	0.908	12	2.0 (0.47–8.4)	0.352
Myometrial invasion ≥50%	120	2.2 (1.2–4.1)	0.016*	83	1.7 (0.47–6.4)	0.404
Lymphovascular space invasion	80	2.3 (1.3–4.1)	0.004**	49	2.9 (1.1–7.7)	0.037*
Adjuvant therapy			0.345			0.032*
None	35	1		33	1	
VBT	129	0.52 (0.15–1.8)	0.293	116	0.41 (0.10–1.7)	0.215
WPRT	47	1.3 (0.39–4.0)	0.698	28	0.092 (0.016–0.54)	0.008**
Chemotherapy	10	1.8 (0.40–8.3)	0.443	7	0.90 (0.15–5.4)	0.910
Chemotherapy and VBT/WPRT	66	1.4 (0.45–4.7)	0.538	34	0.18 (0.038–0.89)	0.035*

Abbreviations: CI, confidence interval; HR, hazard ratio; MMR‐D, mismatch repair deficient; NSMP, no specific molecular profile; VBT, vaginal brachytherapy; WPRT, whole pelvic radiotherapy.

* *p* < 0.05.

** *p* < 0.01.

*** *p* < 0.001.

Stage distribution slightly varied by molecular subgroup in patients receiving whole pelvic radiotherapy (Table S2). For the other treatment modalities, stage distribution was similar between subgroups (Table S2).

To estimate the triage capability of traditional risk factors in stage I MMR‐D and NSMP subtype cancers, we determined the association of age and uterine risk factors with disease‐specific survival separately for the two subgroups (Table S3). Old age was associated with poor survival in the MMR‐D subgroup but not in the NSMP subgroup. Hazard ratios (HR) for deep myometrial invasion and lymphovascular space invasion were fairly similarly increased for the two subgroups, although the HR for deep myometrial invasion in the NSMP subgroup did not reach statistical significance (HR 2.9, 95% confidence interval [CI] 0.83–9.9; *p* = 0.097). Histologic subtype was not associated with outcome in either of the subgroups.

Disease‐specific survival was similar for *MLH1* methylated (n = 154) and MMR‐D nonmethylated (n = 70) cancers (HR 1.5, 95% CI 0.78–3.0; *p* = 0.219), which also held true when the analysis was restricted to stage I MMR‐D cancers (Table S3). Separate multivariable analyses of disease‐specific survival were performed for *MLH1* methylated and MMR‐D nonmethylated cancers (Table 4). Whole pelvic radiotherapy with or without chemotherapy was independently associated with poor survival in MMR‐D nonmethylated but not in *MLH1* methylated cancers. Patients who received no adjuvant therapy or vaginal brachytherapy were chosen as the reference group because of the smaller sample size in these subgroup analyses and because vaginal brachytherapy was not associated with survival in the full MMR‐D cohort (Tables 2 and 3). Of the other variables in the models, deep myometrial invasion and lymphovascular space invasion predicted poor outcome in *MLH1* methylated cancers, whereas old age and lymphovascular space invasion predicted poor outcome in MMR‐D nonmethylated cancers.

**TABLE 4:** Multivariable Cox regression disease‐specific survival analyses for *MLH1* methylated and MMR‐D nonmethylated endometrial cancers

	*MLH1* methylated (n = 154)			MMR‐D nonmethylated (n = 70)		
	N	HR (95% CI)	*p*	N	HR (95% CI)	*p*
Age >65 years	111	1.1 (0.51–2.6)	0.739	28	13 (2.0–93)	0.008**
Stage II–IV	51	1.1 (0.35–3.3)	0.903	17	1.4 (0.27–7.0)	0.698
Histology			0.387			0.730
Endometrioid grade 1–2	110	1		50	1	
Endometrioid grade 3	33	1.8 (0.77–4.4)	0.169	11	0.61 (0.12–3.1)	0.553
Nonendometrioid	11	1.3 (0.41–4.1)	0.659	9	1.9 (0.16–22)	0.623
Myometrial invasion ≥50%	71	3.0 (1.2–7.6)	0.022*	25	0.95 (0.23–3.8)	0.940
Lymphovascular space invasion	48	2.9 (1.3–6.4)	0.010*	18	9.6 (1.6–57)	0.013*
Adjuvant therapy			0.444			0.051
None or VBT	84	1		37	1	
WPRT	28	0.89 (0.25–3.2)	0.851	10	25 (1.6–368)	0.020*
Chemotherapy ± VBT	13	2.5 (0.53–12)	0.249	12	12 (0.56–255)	0.111
Chemotherapy + WPRT	29	1.6 (0.39–6.6)	0.517	11	32 (2.7–388)	0.006**

Abbreviations: CI, confidence interval; HR, hazard ratio; MMR‐D, mismatch repair deficient; VBT, vaginal brachytherapy; WPRT, whole pelvic radiotherapy.

* *p* < 0.05.

** *p* < 0.01.

## 4 DISCUSSION

In this retrospective study, whole pelvic radiotherapy and chemotherapy combined with radiotherapy improved disease‐specific survival in NSMP subtype endometrial cancers, adjusted for age, stage, and high‐risk uterine factors. Although univariable Cox regression analyses indicated that adjuvant therapies are associated with poor disease‐specific survival in the MMR‐D subgroup, this finding disappeared after controlling for appropriate clinicopathologic variables.

Six randomized trials have established the role of adjuvant radiotherapy in decreasing the risk of pelvic and vaginal relapse without improving overall survival in early‐stage endometrial cancer.^16^, ^17^, ^18^, ^19^, ^20^, ^21^ Of the patients in the NSMP subgroup who received adjuvant radiotherapy without chemotherapy in our study, 87.5% (126/144) had stage I cancer. Thus, it seems counterintuitive that whole pelvic radiotherapy improved disease‐specific survival in this molecular subgroup. It should be remembered, however, that the randomized adjuvant therapy trials were conducted prior to the TCGA era. Because molecular subgroups could not be controlled for as confounders of treatment effect, a significant survival advantage in one subgroup may have been obscured. Our findings emphasize the importance of implementing molecular‐integrated risk profile in future clinical trials of adjuvant therapy in endometrial cancer.

Women with early‐stage endometrial cancer are generally stratified to adjuvant therapies on the basis of age and high‐risk uterine factors.^15^ It could be speculated that the triage capability of these traditional risk factors differ for MMR‐D and NSMP subgroups, so that our patients with MMR‐D subtype cancer were not optimally stratified to receive adjuvant treatment. This seems unlikely, however, because old age, deep myometrial invasion, and lymphovascular space invasion were associated with poor disease‐specific survival in stage I MMR‐D subtype cancers.

In an earlier retrospective population‐based cohort study of 535 women with endometrial cancer who received radiotherapy and/or chemotherapy, MMR deficiency was associated with favorable progression‐free survival and overall survival, but on multivariable analysis, the effect of MMR protein status was no longer significant.^22^ In another study, the MMR protein status was not associated with progression‐free survival or overall survival in women treated with adjuvant radiotherapy (n = 66) or chemotherapy (n = 158).^23^ Multivariable analyses were not performed, but in a subgroup analysis of nonendometrioid carcinomas treated with radiotherapy, progression‐free survival, and overall survival were improved in MMR deficient cases compared with MMR proficient cases. On the contrary, MMR proficiency was associated with improved progression‐free survival in chemotherapy‐treated patients with stage III/IV disease.

The design of the study by Reijnen et al. was similar to ours in that they compared separately in MMR proficient and deficient tumor cases the outcome of patients who either received or did not receive adjuvant therapy.^24^ In a sample of 128 women with stage IB/II grade 3 endometrioid carcinoma, adjuvant vaginal brachytherapy/whole pelvic radiotherapy improved disease‐specific survival and overall survival in MMR deficient but not in MMR proficient cases. Although tumors were divided into MMR‐D, p53 abn, *POLE* mutant, and NSMP/p53 wt subgroups according to the ProMisE classifier,^5^ p53 abn, and *POLE* mutant cases were included in the MMR proficient subgroup, unlike in our study.

Discrepant results across studies may be explained by a number of factors, including different study designs and study populations, selection of confounding variables, maturity of follow‐up, and definition of MMR proficiency. Moreover, differences in the categorization of adjuvant therapies may be one factor that explains discrepant findings between our study and the Reijnen study, in which vaginal brachytherapy and whole pelvic radiotherapy were combined into a single treatment group (n = 46 and n = 36 for MMR proficient and deficient cases, respectively).^24^ Namely, we found in the MMR‐D subgroup that the disease‐specific survival was best for patients who received vaginal brachytherapy, worst for those who received whole pelvic radiotherapy, and intermediate for those with no adjuvant therapy (*p* < 0.0005). When vaginal brachytherapy and whole pelvic radiotherapy were combined into one treatment group, the outcome of treated and nontreated women was similar (*p* = 0.957) (Kaplan–Meier plots not shown).

The prognostic value of *MLH1* methylation in endometrial cancer was studied by Shikama et al. who found, after adjustment for confounders, a similar overall survival for MMR‐D nonmethylated and sporadic cases, that is, those with an intact expression of all MMR proteins or loss of MLH1 expression but presence of *MLH1* promoter hypermethylation.^25^ In our earlier study of endometrioid endometrial cancers, we demonstrated worse disease‐specific survival for *MLH1* methylated cases compared with MMR proficient cases.^26^ The present study suggests that *MLH1* methylation status has predictive value in endometrial cancer because whole pelvic radiotherapy and chemotherapy combined with radiotherapy were independently associated with poor disease‐specific survival in MMR‐D nonmethylated but not in *MLH1* methylated tumors. It could be assumed that MMR‐D nonmethylated tumors are inherently resistant to adjuvant therapy especially when high‐risk clinicopathologic factors justify aggressive treatment modalities.

The proportion of Lynch syndrome in our patients with MMR‐D nonmethylated cancer is unknown because germline data were not available. Generally, roughly half of women with MMR‐D nonmethylated endometrial cancer can be identified as having Lynch‐like syndrome which refers to individuals whose tumors show MMR deficiency but who are subsequently found not to carry an MMR gene mutation after germline DNA testing and who also show no evidence of *MLH1* methylation.^27^

Based on their unique prognoses, p53 abn and *POLE* EDM endometrial cancers should be considered distinct from other MMR proficient tumors.^4^ Our study is strengthened by the classification of endometrial cancers into four molecular subgroups based on TCGA^4^ which allowed analyses of NSMP, that is, MMR proficient subgroup from which p53 abn and *POLE* EDM cases are excluded. This study is also strengthened by the confirmation of *MLH1* methylation status in most MMR‐D tumors, relatively high lymphadenectomy rate, consistent adjuvant treatment of patients during the study period, and long follow‐up time. The retrospective analysis of nonrandomized data introduces an obvious limitation and makes this study subject to bias inherent to all studies of similar design, such as unknown confounding variables. As a trade‐off to molecular classification, the size of the MMR proficient (NSMP) subgroup decreased because comprehensive molecular classification could not be performed in 38% (191/508) of cases, mainly due to limited yields of high‐quality DNA from formalin‐fixed paraffin‐embedded (FFPE) samples, and stringent criteria of inclusion for *POLE* sequencing data. Our sequencing failure rate at 26% (114/436) is not atypical for sequencing of FFPE cancer DNA, especially on old tissue.^28^

Intratumoral heterogeneity of protein expression may lead to decreased sensitivity in tissue microarray studies. However, our rate of MMR deficiency was very similar to analyses of whole‐tissue sections.^22^, ^24^ Further, tissue microarrays with two or three core biopsies per tumor block have been shown to adequately represent tumor phenotype, even with antigens known to be heterogeneous.^29^, ^30^ To improve sensitivity, four cores from each block were included in our tissue microarray.^12^ We have earlier demonstrated a high concordance between our tissue microarray and the corresponding whole sections, as concerns expression of L1 cell adhesion molecule, a highly heterogeneous antigen.^12^

In summary, we have demonstrated that the response to adjuvant therapy in endometrial cancer can be predicted by molecular classification and assessment of *MLH1* methylation status. These findings provide an opportunity for the improvement of personalized therapy, and could be applied to clinical trials investigating the use of molecular risk profiles in determining adjuvant therapy in endometrial cancer.

## CONFLICT OF INTEREST

The authors have no conflicts of interest.

## Supporting information

Tables S1‐S3

## Data Availability

All data generated or analyzed during this study are included in this article.
