# Supplementary material for: Mismatch repair protein and MLH1 methylation status as predictors of response to adjuvant therapy in endometrial cancer
Source: Cancer Med. 2021 Jan 15;10(3):1034–42. doi: 10.1002/cam4.3691 (PMC7897956; doi:10.1002/cam4.3691)
Supplement: Supplementary file 1 — Tables S1‐S3 [file CAM4-10-1034-s001.docx]

Supplementary Table S1. Distribution of adjuvant therapies across postoperative ESMO-ESGO-ESTRO risk groups.

| Risk group | N | None | VBT | WPRT | Chemotherapy | Chemotherapy and  VBT/WPRT |
| --- | --- | --- | --- | --- | --- | --- |
| Low | 220 | 54 (24.5%) | 155 (70.5%) | 1 (0.5%) | 4 (1.8%) | 6 (2.7%) |
| Intermediate | 56 | 2 (3.6%) | 47 (83.9%) | 7 (12.5%) | 0 (0%) | 0 (0%) |
| High-intermediate | 62 | 2 (3.2%) | 39 (62.9%) | 15 (24.2%) | 0 (0%) | 6 (9.7%) |
| High | 155 | 6 (3.9%) | 4 (2.6%) | 52 (33.5%) | 8 (5.2%) | 85 (54.8%) |
| Advanced/metastatic | 12 | 4 (33.3%) | 0 (0%) | 0 (0%) | 5 (41.7%) | 3 (25.0%) |

Abbreviations: ESGO, European Society of Gynaecological Oncology; ESMO, European Society for Medical Oncology; ESTRO, European Society for Radiotherapy and Oncology; VBT, vaginal brachytherapy; WPRT, whole pelvic radiotherapy.

Supplementary Table S2. Distribution of adjuvant therapies among MMR-D (n = 287) and NSMP (n = 218) molecular subgroups.

| Adjuvant therapy | Subgroup | Stage I | Stage II | Stage III | Stage IV | P |
| --- | --- | --- | --- | --- | --- | --- |
| None | MMR-D | 31 (10.8%) | 1 (0.3%) | 3 (1.0%) | 0 (0%) | 0.111 |
|  | NSMP | 29 (13.3%) | 1 (0.5%) | 0 (0%) | 3 (1.4%) |  |
| VBT | MMR-D | 129 (44.9%) | 0 (0%) | 0 (0%) | 0 (0%) | – |
|  | NSMP | 116 (53.2%) | 0 (0%) | 0 (0%) | 0 (0%) |  |
| WPRT | MMR-D | 28 (9.8%) | 17 (5.9%) | 2 (0.7%) | 0 (0%) | 0.047 |
|  | NSMP | 10 (4.6%) | 18 (8.3%) | 0 (0%) | 0 (0%) |  |
| Chemotherapy | MMR-D | 3 (1.0%) | 1 (0.3%) | 3 (1.0%) | 3 (1.0%) | 0.678 |
|  | NSMP | 3 (1.4%) | 0 (0%) | 3 (1.4%) | 1 (0.5%) |  |
| Chemotherapy and  VBT/WPRT | MMR-D | 15 (5.2%) | 3 (1.0%) | 47 (16.4%) | 1 (0.3%) | 0.329 |
|  | NSMP | 7 (3.2%) | 4 (1.8%) | 21 (9.6%) | 2 (0.9%) |  |

Abbreviations: MMR-D, mismatch repair deficient; NSMP, no specific molecular profile; VBT, vaginal brachytherapy; WPRT, whole pelvic radiotherapy.

Supplementary Table S3. Univariable Cox regression disease-specific survival analyses for stage I MMR-D and NSMP subtype endometrial cancers.

|  | MMR-D (n = 206) | | | NSMP (n = 165) | | |
| --- | --- | --- | --- | --- | --- | --- |
|  | N | HR (95% CI) | P | N | HR (95% CI) | P |
| Age >65 years | 122 | 3.1 (1.2-8.4) | 0.023* | 90 | 2.1 (0.54-8.2) | 0.280 |
| Histology  Endometrioid grade 1-2  Endometrioid grade 3  Nonendometrioid | 170  26  10 | 1  2.1 (0.80-5.8)  0.97 (0.13-7.3) | 0.314  0.131  0.977 | 157  4  4 | 1  6.1 (0.77-49)  0 – not calculable | 0.232  0.087  0.987 |
| Myometrial invasion ≥50% | 66 | 4.2 (1.8-9.6) | 0.001** | 42 | 2.9 (0.83-9.9) | 0.097 |
| Lymphovascular space invasion | 39 | 8.9 (3.9-20) | 0.0000002*** | 26 | 3.6 (1.0-13) | 0.049* |
| *MLH1* methylated ^a^ | 103 | 1.8 (0.59-5.6) | 0.298 | N/A | | |

Abbreviations: CI, confidence interval; HR, hazard ratio; MMR-D, mismatch repair deficient; NSMP, no specific molecular profile.

^a^ Reference category: MMR-D nonmethylated (n = 53).

* P < 0.05

** P < 0.01

*** P < 0.001
